# Cascade Upgrading of Biomass-Derived Furfural to γ-Valerolactone Over Zr/Hf-Based Catalysts

**DOI:** 10.3389/fchem.2022.863674

**Published:** 2022-03-07

**Authors:** Wenjuan Sun, Haifeng Li, Xiaochen Wang, Anqiu Liu

**Affiliations:** ^1^ School of Chemistry and Materials Science, Ludong University, Yantai, China; ^2^ School of Energy Materials and Chemical Engineering, Hefei University, Hefei, China

**Keywords:** biomass, furfural, γ-valerolactone, Zr/Hf-based catalysts, active site regulation

## Abstract

Biomass feedstocks are promising candidates of renewable clean energy. The development and utilization of biological energy is in line with the concept of sustainable development and circular economy. As an important platform chemical, γ-valerolactone (GVL) is often used as green solvent and biofuel additive. Regarding this, the efficient synthesis of GVL from biomass derivative furfural (FF) has attracted wide attention recently, However, suitable catalyst with appropriate acid-base sites is required due to the complex reaction progress. In this *Mini Review*, the research progress of catalytic synthesis of GVL from furfural by Zr/Hf-based catalysts was reviewed. The different effects of Lewis acid-base and Brønsted acid sites in the catalysts on each steps in the reaction process were discussed firstly. Then the effects of regulation of acid-base sites in the catalysts was also studied. Finally, the advantages and challenges of Zr/Hf-based catalysts in FF converted to GVL system were proposed.

## Introduction

Although the exploration and utilization of fossil energy promote the development of human society, it also causes nonnegligible harm to the environment, which makes people focus on available energy to reduce dependence on fossil energy (Roman-Leshkov et al., 2007; Luterbacher et al., 2014; Zhang et al., 2019). Biomass, as the only renewable organic carbon source, has received extensive attention due to its abundance, cheapness, and availability (Li et al., 2014; Zhao et al., 2019; Li H. et al., 2020). A variety of valuable compounds (e.g., xylose, furfural, furfuryl alcohol, levulinic acid and its esters, and γ-valerolactone) can be obtained from biomass (Liu et al., 2015; Li F. et al., 2017; Li H. et al., 2017; Lingaiah, 2018; Li et al., 2019a; Luo et al., 2019). Among them, γ-valerolactone (GVL) has excellent physical and chemical properties such as high boiling point (207°C), low melting point (31°C), and low toxicity (LD_50_ = 8,800 mg/kg). It can be used as a green organic solvent in a variety of reactions, and has broad application prospects in the organic synthesis, biorefinery, and food industry (Yan et al., 2015; Ye et al., 2020b). In addition, GVL can be further converted into various valerate esters (these have been identified as new generation biofuels), which can be used to synthesize various biomass-based liquid fuels (Yu et al., 2019).

In recent years, the related research on the synthesis of GVL mainly focuses on the direct hydrogenation or catalytic transfer hydrogenation (CTH)with levulinic acid and its esters as substrates. Both noble metal (Ru, Rh, Pt, Pd, Au) catalysts and non-precious metal (Ni, Cu, Co.) catalystshave been used for the hydrogenation of LA to GVL (Yuan et al., 2013; Luo et al., 2014; Molleti et al., 2018). Obregon et al. studied the liquid phase hydrogenation of LA on Ni/Al2O3, after reacting at 250°C and 6.5 MPa H2 pressure for 2 h, The yiled of GVL reached 92%I. (Obregon et al., 2014). Although high yield was achieved by hydrogenation, however, the use of pressur-ized-hydrogen gas is often associated with potential explosion hazard, so the transfer hydrogenation strategy for the synthesis of GVL from LA has been developed. Numerous sup-ported Ru, Pd, Ni, and Cu catalysts were investigated to this reaction (Dutta et al., 2019; Ye et al., 2020b). Fu et al. firstly reported an non-precious skeletal Ni catalyst which could effective catalyze the reaction with i-PrOH as H-donor at room temperature over 9 h (Yang, et al., 2013). In addition, different hydrogen donors such as formic acid, hydrosilicon and alcohol have been exploited for this transformation, compare to other H-donors, the secure, safe and easily operated alcohol not only can act as H-donor, but also can serve as a solvent, furthermore, it can enhance the selec-tivity in the hydrogenation process, too(He et al., 2020a).Compared with levulinic acid, furfural (FF) is more available from biomass feedstocks, so the researchers considered FF directly as a feedstock for GVL production (Bui et al., 2013).

**FIGURE 1:**
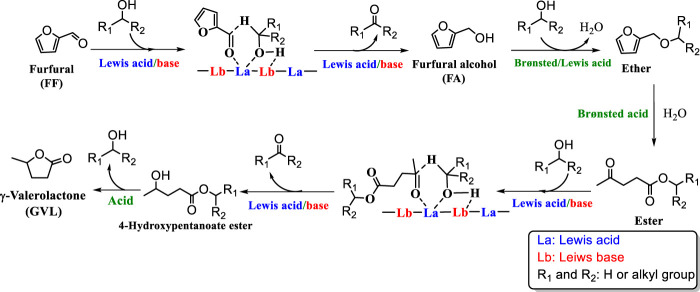
Possible reaction mechanism for the cascade conversion of biomass-derived furfural (FF) to γ-valerolactone (GVL).

The conversion of FF to GVL requires a series of cascade reactions (Figure 1) such as CTH, etherification, ring-opening, partial hydrogenation, and cyclization reaction (Zhu et al., 2016). Such complex reaction processes require higher performance catalysts. Therefore, it is necessary to fully consider both the structure and acid-base properties of the catalyst to improve the catalyst activity. Since Zr/Hf-based catalysts show excellent catalytic performance in CTH reactions and are more economical than precious metals, more and more researchers applied them to the reaction of converting GVL from FF (Li et al., 2016; Wu et al., 2018; Zhou et al., 2019a; Wang et al., 2019).

At present, some excellent reviews are related to the synthesis of GVL, but most of these reviews focus on the synthesis of GVL with levulinic acid and its esters as the substrate (Dutta et al., 2019; Ye et al., 2020b). In this mini-review, the latest progress in the design of high-performance Zr/Hf-based catalysts for GVL production from FF. Some variables affecting the design of Zr/Hf-based catalysts such as the regulation of active sites of catalysts and the physical and chemical properties of catalysts were summarized. In addition, the reaction parameters in regulating conversion efficiency was discussed, providing insights for the development of efficient, economic, and sustainable catalytic systems that would be important for future research.

## Effects of Catalyst Properties on the Synthesis of GVL From FF

In the system of FF for the synthesis of GVL, Zr/Hf-based catalysts showed good performance, as shown in Table 1. Zhu et al. first used Au/ZrO_2_ (providing Lewis acid-base sites) with ZSM-5 (providing Brønsted acid sites) to catalyze the conversion of FF to GVL (Zhu et al., 2016). The experimental results showed that when Au/ZrO_2_ was used as the catalyst, FF was almost completely converted to furfuryl alcohol (FA) (99.0% yield) rather than GVL. Similarly, no GVL was detected when only ZSM-5 was used as the catalyst. These results showed that the presence of both Lewis acid-base and Brønsted acid sites in the catalyst was necessary to successfully catalyze the conversion of FF to GVL. Rojas-Buzo et al. found that the prepared Hf-MOF-808 catalyst could successfully catalyze the CTH reaction of FF to FA and levulinic acid to obtain GVL, but could not directly catalyze the synthesis of GVL from FF(Rojas-Buzo et al., 2018). However, when the Hf-MOF-808/Al-β zeolite (containing Brønsted acid sites) combined catalyst was applied to this reaction, a good 75% yield of GVL was obtained at 120°C for 48 h. This result strongly shows that Brønsted acid is crucial to the ring-opening process involved in the conversion of FA to levulinate in this reaction process. Although combined catalyst system could improve the reaction yield, the catalyst preparation process becomes complicated and the production cost increases. To simplify the preparation process of the catalyst and increase reaction yield of GVL, the exploration of bifunctional catalysts containing both Lewis and Brønsted acid sites has attracted more and more attention. Bui et al. first used the physical mixture of Zr-Beta and mesoporous Al-MFI zeolite as Lewis acid and Brønsted acid catalysts to convert FF into GVL in one-pot (Bui et al., 2013). Later, Iglesias et al. synthesized a bifunctional catalyst containing both Lewis acid and Brønsted acid by loading ZrO_2_ on SBA-15 zeolite (Iglesias et al., 2018). The catalyst can control the strength of Lewis acid and Brønsted acid by changing the number of ZrO_2_ layers. Kinetic studies showed that the strength of Lewis acid in the catalyst had an important influence on the distribution of products. Strong Lewis acid sites promote etherification and isomerization of FA rather than MPV reduction. Srinivasa Rao et al. used the impregnation method to load different proportions of ZrO_2_ and phosphotungstic acid (TPA) on β-zeolite to further study the effect of Lewis/Brønsted acid content in the catalyst on the yield of GVL (Srinivasa Rao et al., 2021). The experimental results show that more Brønsted acid sites and fewer Lewis acid sites in the catalyst are more conducive to the production of levulinic acid ester rather than GVL. Therefore, the key to obtain high yield GVL is to control the Lewis acid-base and Brønsted acid sites with appropriate strength and number of bifunctional catalysts. Very recently, Tan et al. synthesised a variety of novel coordination organophosphate–Hf polymers from vinylphosphonic acid (VPA), *p*-toluenesulfonic acid (*p*-TSA), and HfCl. Specifically, VPA–Hf(1 : 1.5)-0.5 with an appropriate L/B acid ratio of 5.3 and was found to exhibit superior performance in the one-step conversion of furfural (FF) to γ-valerolactone (GVL) in a high yield of 81.0%, with a turnover frequency of 5.0 h^−1^. (Tan et al., 2022).

**TABLE 1:** Catalytic production of γ-valerolactone (GVL) from furfural (FF)over Zr/Hf-based catalysts.

Entry	Catalysts	Acidity (mmol/g)	L/B	H-donor	Adjustment of active sites	Reaction conditions	GVL yield (%)	Ref
1	Zr-Beta + Al-MFI-ns	--	--	2-butanol	Lewis acid site and Brønsted acid site are independent of each other, which can adjust the content and strength of Lewis acid and Brønsted acid in the catalyst respectively	120°C, 48 h	78	Bui et al. (2013)
2	Au/ZrO2+ZSM-5	--	--	2-propanol		120°C, 30 h	80.4	Zhu et al. (2016)
3	Hf-MOF 808+Al-β zeolite	--	--	2-propanol		120°C, 48 h	75	Rojas-Buzo et al. (2018)
4	ZrO2-SBA-15(2)	0.32	0.08	2-propanol	With the increase of the number of ZrO2 film layers supported on the surface of SBA-15, the strength of Lewis acid in the catalyst increases, while the strength of Brønsted acid decreases	170°C, 7 h	37	Iglesias et al. (2018)
5	Zr-KIT-5	1.86	6.5	2-propanol	Change the loading of Zr in the catalyst	180°C, 6 h	40.1	He et al. (2020b)
6	HZ-ZrP 1-5	0.87	4.1	2-propanol	Change the ratio of zeolite and NH4H2PO4 in the catalyst	185°C, 18 h	64.2	Ye et al. (2020a)
7	HPW/Zr-Beta	0.78	3.2	2-propanol	Use different acid treatment catalysts	160°C, 24 h	68	Winoto et al. (2019)
8	20%Zr-5%T-zeolite	1.67	1.53	2-propanol	Adjust the ratio of TPA and Zr in the catalyst	170°C, 10 h	90	Srinivasa Rao et al. (2019)
9	DUT-67(Hf)-0.06	1.28	--	2-propanol	Treatment of DUT-67-(Hf) with different concentrations of sulfuric acid	180°C, 8 h	70.7	Li et al. (2019b)
10	FM-Zr-ARS	0.55	0.23	2-propanol	Modification of the catalyst with formic acid	160°C, 8 h	72.4	Peng et al. (2021)
11	ZPS-1.0	--	3.25	2-propanol	Change the amount of Zr in the catalyst	150°C, 18 h	80.4	Li et al. (2021c)

Zeolite with a complex microporous structure has an open framework with regular pore size and appropriate size, which is conducive to mass transfer and is easy to adjust acidity (Wang et al., 2017; Wang et al., 2020; Peng et al., 2020; Chai et al., 2021). Since zeolite has these unique advantages, the existing catalysts for FF conversion to GVL are mostly prepared with various zeolites as supporter. These catalysts mainly change the content of Lewis acid sites in the catalysts by changing the metal loading, and different kinds and concentrations of acids are used to control the content of Brønsted acid in the catalysts (Srinivasa Rao et al., 2019; Winoto et al., 2019; He et al., 2020a; Ye et al., 2020a). He et al. adjusted the content of Lewis/Brønsted acid in the catalyst by adding different amounts of ZrOCl_2_·8H_2_O(He et al., 2020b). The more Zr is loaded in the catalyst, the higher the molar ratio of Lewis acid to Brønsted acid is. NH_3_-TPD results showed that with the increase of Zr loading in the catalyst, the total number of acid sites in the catalyst increased gradually. But excessive Zr loading will produce zirconia clusters, which will reduce the activity of the catalyst. Li et al. treated the catalyst by soaking DUT-67 (Hf) in different concentrations of sulfuric acid solution to change the content of Brønsted acid (Li W. et al., 2019). The results showed that with the increase of sulfuric acid concentration, the total content of acid sites in the catalyst increased continuously, but excessive Brønsted acid in the catalyst would lead to side reactions, which decreased the yield of GVL. SrinivasaRao et al. loaded phosphotungstic acid (TPA) and ZrO_2_ with different contents inside and outside the pores of SBA-15, respectively (Srinivasa Rao et al., 2021). Under the premise of keeping the total Lewis acid content in the catalyst unchanged, the molar ratio of Lewis acid to Brønsted acid in the catalyst was adjusted by controlling the amount of ZrO_2_ and TPA. The catalyst showed excellent catalytic activity, and the yield of GVL reached 90% at 170°C for 10 h.

In addition to using zeolite as a carrier, bifunctional materials prepared with ligands base on biomass derivatives are also applied to the conversion of FF to GVL. Using alizarin red S (ARS) as the ligand, Peng et al. synthesized FM-Zr-ARS catalyst by a simple hydrothermal method (Peng et al., 2021). The sulfonic acid group contained in ARS can acted as Brønsted acid for the ring-opening reaction, however, it cannot effectively regulate the relative content of different active sites in the catalyst. As a key step in the conversion of FF to GVL, CTH reaction is generally completed through a six-membered ring transition state. Lewis acid sites are usually used to activate H on the aldehyde group and C connected with the alcohol hydroxyl group. Lewis base sites are mainly used to activate the alcohol hydroxyl group, making H easier to remove. Finally, the transfer hydrogenation process is completed through the six-membered ring transition state (Li et al., 2018; Zhou et al., 2019b; Li et al., 2019c). Jarinya et al. found that Hf-UiO-66 has lower activation energy (13.5 kcal/molvs 14.9 kcal/mol) than Zr-UiO-66 based on density functional theory (DFT)(Sittiwong et al., 2021). It is due toHf having stronger Lewis acidity, Hf has better performance than Zr in CTH reaction under the same preparation conditions (Luo et al., 2014; Xie et al., 2016; Injongkol et al., 2017; Li X. et al., 2020). Tan et al. prepared a new coordination organic phosphate-Hf polymer VPA-Hf(1:1.5)-0.5, which showed good activity for one-pot cascade conversion of FF to GVL. By controlling the ratio of vinyl phosphoric acid, *p*-toluenesulfonic acid and HfCl_4_, the content of Lewis acid sites and B acid sites can be accurately adjusted, and the E factor value (0.19) shows that the conversion process mediated by the catalyst is ecologically friendly.

## Effect of Reaction Parameters

The reaction can be carried out under mild conditions (120°C) when combined catalysts were used (Table 1). For bifunctional catalysts containing both Lewis acid and Brønsted acid, although the preparation of the catalyst is simpler and the cost is reduced, a higher reaction temperature (150–180°C) is often required to ensure the sufficient progress of the reaction. This may due to the independent active sites can also effectively reduce the adverse effects of steric hindrance in the reaction process, so the reaction can be carried out under mild conditions. However, the disadvantages such as excessively long reaction time and more tedious catalyst preparation process cannot be ignored. The key CTH reactions in the reaction process are completed by MPV reduction reaction, and more green and safe alcohols are usually used as H-donors to avoid the use of dangerous high-pressure H_2_and corrosive formic acid. In general, the β-H of secondary alcohols is easier to be removed from the transition state, so the hydrogen supply capacity of secondary alcohols is stronger than that of primary alcohols (Elsayed et al., 2020; Li J. et al., 2021). However, the steric hindrance of secondary alcohols will gradually increase with the extension of the carbon chain, and excessive steric hindrance is not conducive to the formation of stable transition states, thereby reducing the hydrogen supply capacity (Li M. et al., 2021; Li W. et al., 2021). Therefore, due to the small steric hindrance, 2-propanol was used as the H-donor to prepare GVL in most cases. In addition, the reusability of the catalyst is also an important aspect to evaluate the catalytic system. However, humus is usually formed during the reaction, which not only affects the carbon balance of the reaction system but also reduces the activity of the catalyst during recycling. Usually, calcination can remove the humus attached to the catalyst and restore the activity of the catalyst (Iglesias et al., 2018; Ye et al., 2020a; Tang et al., 2021). In addition, the catalyst may also have active site leaching during recycling, and it needs to be treated with acid before being put into the next recycling (Li W. et al., 2019).

## Conclusion and Outlook

GVL is an important biomass derivative, which can be used as green solvents and biofuels. Highly efficient cascade conversion of FF to GVL presents great challenges due to complex reaction processes and high requirements for catalyst performance. In this mini-review, the influence of the catalyst preparation process on catalyst activity was reviewed, and the reaction parameters such as temperature and H- donor were also discussed. The acid-base properties of the catalyst have a great influence on its catalytic performance. The Lewis acid-base sites in the catalyst are mainly used to catalyze the CTH reaction, and the crucial ring-opening reaction needs to be carried out in the presence of Brønsted acid sites. There is no doubt that higher acid content in the catalyst can provide more active sites, but the imbalance of Lewis acid and Brønsted acid ratio can easily lead to undesirable side reactions. It may lead to carbon imbalance and GVL yield reduction, while the formation of humus attached to the catalyst will reduce the reusability of the catalyst.

Renewable biomass-based carbonaceous support catalysts have great potential for the green synthesis of GVL. Organic hybrid materials have proved to have good activity for CTH reaction, but the Brønsted acid sites are usually not sufficient to catalyze the ring-opening reaction. Therefore, how to improve the strength of Brønsted acid while ensuring the stability of the catalyst structure is the challenge that must be overcome for its application for FF synthesis to GVL. In addition, the accurate control of the strength and content of each active site in the catalyst can better control the reaction process, which is crucial to improving the yield of GVL. Due to the strong Lewis acidity of Zr/Hf materials, Zr/Hf-Based Catalysts showed high performance in the reaction of convert FF to GVL. However, most of the exiting catylic system still suffered from high temperature as well as not so excellent yield, so it is still a challegen to design novel and effecient catalyst for this reaction.
